# Isogarcinol Is a New Immunosuppressant

**DOI:** 10.1371/journal.pone.0066503

**Published:** 2013-06-13

**Authors:** Juren Cen, Mingshu Shi, Yanfang Yang, Yanxia Fu, Hailing Zhou, Mengqi Wang, Zhenyi Su, Qun Wei

**Affiliations:** 1 Department of Biochemistry and Molecular Biology, Beijing Normal University, Gene Engineering and Biotechnology Beijing Key Laboratory, Beijing, P. R. of China; 2 Province and Ministry Established Key Laboratory of Resource and Complex Prescription of Traditional Chinese Medicine, Hubei University of Chinese Medicine, Wuhan, P. R. of China; IIT Research Institute, United States of America

## Abstract

Calcineurin (CN), a unique protein phosphatase, plays an important role in immune regulation. In this study we used CN as a target enzyme to investigate the immunosuppressive properties of a series of natural compounds from *Garcinia mangostana* L., and discovered an active compound, isogarcinol. Enzymatic assays showed that isogarcinol inhibited CN in a dose-dependent manner. At concentrations resulting in relatively low cytotoxicity isogarcinol significantly inhibited proliferation of murine spleen T-lymphocytes induced by concanavalin A (ConA) and the mixed lymphocyte reaction (MLR). In addition, it performed much better in acute toxicity tests and via oral administration in mice than cyclosporin A (CsA), with few adverse reactions and low toxicity in experimental animals. Oral administration of isogarcinol in mice resulted in a dose-dependent decrease in delayed type hypersensitivity (DTH) and prolonged graft survival in allogeneic skin transplantation. These findings suggest that isogarcinol could serve as a new oral immunomodulatory drug for preventing transplant rejection, and for long-term medication in autoimmune diseases.

## Introduction

The phosphorylation and dephosphorylation of signal transduction proteins play critical roles in cellular immune responses. Calcineurin (CN), a unique calcium/calmodulin activated protein serine/threonine phosphatase [1], [2], is a heterodimer composed of a 61 kD catalytic subunit (CNA) and a 19 kD regulatory subunit (CNB). It is involved in a variety of physiological and pathological processes including T-cell activation, apoptosis, neurodegenerative disease, and cardiac hypertrophy [3].

Clinical interest in CN has focused on T-lymphocytes and other immune cells, especially on the CN - nuclear factor of activated T cells (NFAT) signaling pathway, in which CN is activated by binding of Ca^2+^ to calmodulin. The activated calcineurin dephosphorylates NFAT, leading to its nuclear localization and activation of expression of its target genes [3], [4], [5], [6], [7], [8]. The immunosuppressive drugs cyclosporin A (CsA) and tacrolimus (also called FK506), which inhibit calcineurin activity when complexed with their specific cytoplasmic receptors (cyclophilin and FK506-binding protein (FKBP), respectively) [9], [10], [11], [12], have revolutionized transplantation medicine. However their therapeutic effects are limited in large part by their side effects, including nephrotoxicity and neurotoxicity [13], and therefore the development of new immunosuppressive drugs is highly desirable. CN generally serves as the target enzyme for screening potential immunosuppressive drugs.

Natural products have played an important role in the discovery and development of drugs. Many tropical plants have been evaluated as potential therapeutic agents. *Garcinia mangostana* L., also known as the mangosteen or the purple mangosteen, is a tropical evergreen tree that belongs to the family Guttiferae and is found extensively in southeast Asia [14]. People in this area have long used the pericarp (peel, rind, and hull) or the ripe fruit of mangosteen as a traditional medicine to treat abdominal pain and wound infection [15]. Moreover, mangosteen also has anti-inflammatory activity [16].

We have previously investigated the immunosuppressive properties of natural products; especially the diverse group of CN inhibitors which have immune regulatory activity both directly and indirectly. We found that kaempferol, quercetin and glycyrol could inhibit calcineurin activity and had immunosuppressive activity and thus could act as novel immunomodulatory drugs [17], [18], [19], [20], [21], [22].

In this study we investigated a series of natural compounds from *Garcinia mangostana* L. in an attempt to find a novel oral immunosuppressant with low toxicity. In a preliminary study, we found that ethanol extracts could inhibit calcineurin activity, and in the present work we identified a component responsible for strong inhibition of CN activity using a CN enzymatic assay. Then, to explore its immunomodulatory activity, we investigated its effect on the proliferation of ConA-stimulated murine spleen lymphocytes, the *in vitro* mixed lymphocyte reaction (MLR), delayed type hypersensitivity (DTH), and *in vivo* allogeneic skin graft rejection.

## Materials and Methods

### Ethics statement

The experimental procedures were approved by the Animal Ethics Committee of Beijing Normal University and were carried out in strict accordance with institutional guidelines. All efforts were made to minimize the number of animals used and their suffering.

### Materials

*Garcinia mangostana* L. (shan zhu zi) was obtained from Chengmai County, Hainan province, China. *E. coli* BL21 (DE3) containing rat CNA cDNA was from our laboratory. p-Nitrophenyl phosphate (p-NPP), BSA, DTT and concanavalin A (Con A) were from Sigma–Aldrich (St. Louis, MO), and a Cell Counting Kit-8 (CCK-8) was from Dojindo Molecular Technologies, Inc. (Beijing). C57BL and BALB/c mice (6–8 weeks) were purchased from the Vital River Laboratories (Beijing, China). Ethanol, petroleum ether, ethyl acetate, n-butanol, acetone, acetic acid and 2,4-dinitrofluorobenzene (DNFB) were purchased from Beijing Chemical Reagent Company. Silica gel chromatography equipment was from Qingdao Marine Chemical Plant. Dimethyl sulfoxide (DMSO) was obtained from Amethyst Chemicals and RPMI 1640 medium from macgene. CsA was from Shanghai Pureone Biotechnology and Peanut oil from Shandong Luhua group Co.,Ltd.

### Expression and purification of CNA

CNA was expressed and purified as described previously [23]. Protein purity was analyzed by SDS-PAGE.

### Extraction and isolation of compounds for screening

Air-dried bark of *Garcinia mangostana* L. was ground into powder and extracted with 75% ethanol. The concentrated extract was dispersed in water and partitioned successively with petroleum ether, ethyl acetate (EtOAc) and n-butanol (n-BuOH). The EtOAc fraction was subjected to silica gel chromatography (100–200 mesh) using a solvent of petroleum ether and acetone (6∶1∼1∶1, v/v), to yield 15 fractions (A∼O). CN activity assays were performed to determine the inhibitory effect of different fractions. Fraction M, the most active fraction, was subjected to silica gel chromatography using a solvent of petroleum ether, acetone and acetic acid (2∶1: 3%∼1∶1: 3%), to yield 7 fractions (M1∼M7). Fraction M3, the most active fraction , was then recrystallized with 95% ethanol to yield the component designated compound I.

### Compound I identification

Compound I was identified as isogarcinol using ^1^HNMR, ^13^CNMR and ESI-MS spectral analysis and comparison with data in the literature.

### CN activity assays

CN activity assays were performed using p-NPP as substrate in 1 mM CaCl_2_, 0.5 mM MnCl_2_, 2 mM CaM, 2 mM CNB, 1 mM DTT, 0.1 mg/ml BSA, and 50 mM Tris-HCl (pH 7.4) at 4**°**C. After addition of CNA, solutions were pre-incubated for 10 min at 4**°**C before initiating the reaction by adding p-NPP to a final concentration of 20 mM. A sample not containing Compound I was used as matched control. Enzyme activity was monitored with a spectrophotometer, measuring absorbance at 410 nm. Phosphatase activities are presented as percentages of control activity.

### Cell viability assay

Cell viability was measured using the tetrazolium salt-based CCK-8 assay. Isogarcinol and CsA dissolved in DMSO were diluted with medium to high concentration stored solvent. Spleen lymphocytes were seeded into 96-well plates at 1.5×10^7^ cell/mL in 100 µL complete medium; then RPMI 1640 medium containing various concentrations of isogarcinol or CsA was added to a final volume of 200 µL. The final concentrations of isogarcinol were 0.83, 4.15, 8.31, 12.46, 20.76 and 41.53 µM, and those of CsA were 1, 5, 10, 15, 20, 30 and 40 µM. The highest content of DMSO was 0.1%. A control reaction received RPMI 1640 medium containing only 0.1% DMSO. Plates were incubated for 68 h at 37°C in 5% CO_2_, then 20 µL of CCK-8 reagent was added to the wells and incubated for 4 h. Absorbance at 450 nm was measured on an ELISA reader [24]. The experiment was repeated five times. Cell viability was calculated using the following equation: viability (%) = (OD450 of isogarcinol group/OD450 of control group)×100.

### Con A-induced mouse splenocyte proliferation

Spleen lymphocytes were seeded in 96-well plates at 5×10^6^ cell/mL in 100 µL complete medium; then concanavalin A (Con A) and RPMI 1640 medium containing various concentrations of isogarcinol were added to a final volume of 200 µL. The final concentrations of isogarcinol were 0.83, 4.15, 8.31, 12.46, and 20.76 µM. The highest content of DMSO was 0.1%.The control group was treated with RPMI 1640 medium containing only 0.1% DMSO. After 24, 48 and 72 h of incubation, proliferation was measured using the CCK-8 assay as described above. Cell growth inhibition was calculated using the following equation: inhibition (%) = [(OD450 of control group – OD450 of isogarcinol group)/OD450 of control group]×100.

### Mixed lymphocyte reaction

A unidirectional MLR was performed with five replicates as previously described [19]. BALB/c splenocytes (spBALB) treated with mitomycin C (50 mg/ml, Sigma) at 37°C for 50 minutes served as stimulators and C57BL/6 (spC57) splenocytes served as responders. Both stimulators and responders were seeded into 96-well plates at a density of 1×10^7^ cells/well, along with different isogarcinol concentrations and RPMI 1640 medium with 0.1% DMSO instead of isogarcinol served as control. The final concentrations of isogarcinol were 0.83, 4.15, 8.31, 12.46, and 20.76 µM. The highest content of DMSO was 0.1%. After 48, 72 and 96 h of incubation, proliferation was measured using the CCK-8 assay as described above.

### Toxicity test

An acute toxicity test was performed in mice using the fixed-dose procedure, which is a sequential testing scheme proposed by the British Toxicology Society [25]. Since mice treated with isogarcinol at an initial dose of 500 mg per kg body weight did not die, an acute toxicity test was carried out. Sixteen mice, eight of each sex, were divided into two groups. Isogarcinol was administered orally at a dose of 500 mg/kg to one group and 2000 mg/kg to the other. Isogarcinol dissolved in DMSO was diluted with peanut oil. The highest content of DMSO was 5%. The mice were observed for two weeks to assess any changes in reactivity, gait, motor activity, respiration rate, etc., and especially death.

To further investigate the effect of isogarcinol in animals, BALB/c mice were randomly divided into the following three groups (eight per group): control group, CsA group and isogarcinol group. Mice in the control group received an oral gavage of 0.2 mL peanut oil containing 2% DMSO daily for 4 days, mice in the CsA group received the same volume containing 500 mg/kg CsA on the first day and 100 mg/kg on the following three days, and mice in the isogarcinol group received the same volume containing 500 mg/kg isogarcinol on the first day and 100 mg/kg on the following three days. CsA and isogarcinol dissolved in DMSO were diluted with peanut oil. The content of DMSO was 2%. Blood was taken and assays were performed to determine levels of glutamic-pyruvic transaminase, glutamic-oxalacetic transaminease, total bilirubin, urea nitrogen, and serum creatinine.

### DNFB induced DTH assay

A DTH assay was carried out as previously described [26], [27]. Male BALB/c mice were randomly divided into the following six groups (eight per group): negative control group, model group, CsA group, and three experimental isogarciol groups. Mice in the negative control and model groups received an oral gavage of 0.2 mL peanut oil containing 2% DMSO daily for 6 days, mice in the CsA group received the same volume containing 40 mg/kg CsA daily for 6 days, and mice in the three experimental groups received the same volume containing 40, 70 and 100 mg/kg isogarcinol, respectively, daily for 6 days. CsA and isogarcinol dissolved in DMSO were diluted with peanut oil. The content of DMSO was 2%. Fur was removed from all mice (2 cm×2 cm) to fully expose the abdomen. They were initially sensitized by uniformly painting 50 µl 2% 2,4-dinitrofluorobenzene (DNFB) dissolved in acetone and peanut oil (1∶1) on the shaved abdomens on days 1 and 2. In the negative control group 50 µl solvent without DNFB was used. Five days after the second sensitization, all mice were challenged with 10 µl 2% DNFB on both sides of their right ear, and the left ears were treated with solvent alone. They were killed 24 h after challenge and the spleen and thymus were rapidly removed and weighed. The spleen and thymus indexes were expressed as spleen weight (mg)/10 g weight mouse and thymus weight (mg)/10 g weight mouse, respectively. Ear swelling was expressed as the difference between the weights of the left and right ear patches obtained from 8-mm punches 24 h after challenge. The punches were obtained in a blinded manner.

### Allogeneic skin transplantation

Skin transplantation was performed following a protocol reported previously [19]. Briefly, C57BL/6 donor mice were killed and abdominal skin was removed for syngeneic allogeneic grafts. Full thickness abdominal skin grafts of approximately 1 cm^2^ were sutured onto graft beds prepared on the posterior thorax of euthymic BALB/c recipient mice. The grafts were covered with albolene gauze bandages and examined every other day from 7 days after transplantation. To examine grafts, mice were lightly anesthetized with pentobarbital sodium, and the bandages were removed. They were replaced after examination. After making the skin allografts, isogarcinol (100 mg/kg), CsA(40 mg/kg) or control (solution containing 2% DMSO and peanut oil) was administered orally daily until rejection. CsA and isogarcinol dissolved in DMSO were diluted with peanut oil. The content of DMSO was 2%. The health of donor skin grafts was monitored by visual and tactile inspection. The day of skin rejection was defined as the day graft necrosis reached approximately 80%. Graft rejection is expressed as median survival time (MST) ± SEM.

### Statistical analysis

All experiments were performed in at least triplicate and unless otherwise stated the results are expressed as means ± SD. Statistical significance was determined using SPSS 11.0 software (SPSS, Chicago, IL, USA). The students t-test was used for paired samples, and differences were deemed significant at three levels: p<0.001 (***), p<0.01 (**), and p<0.05 (*).

## Results

### Inhibition of CN activity by *Garcinia mangostana* L. fractions

**Figure 1:**
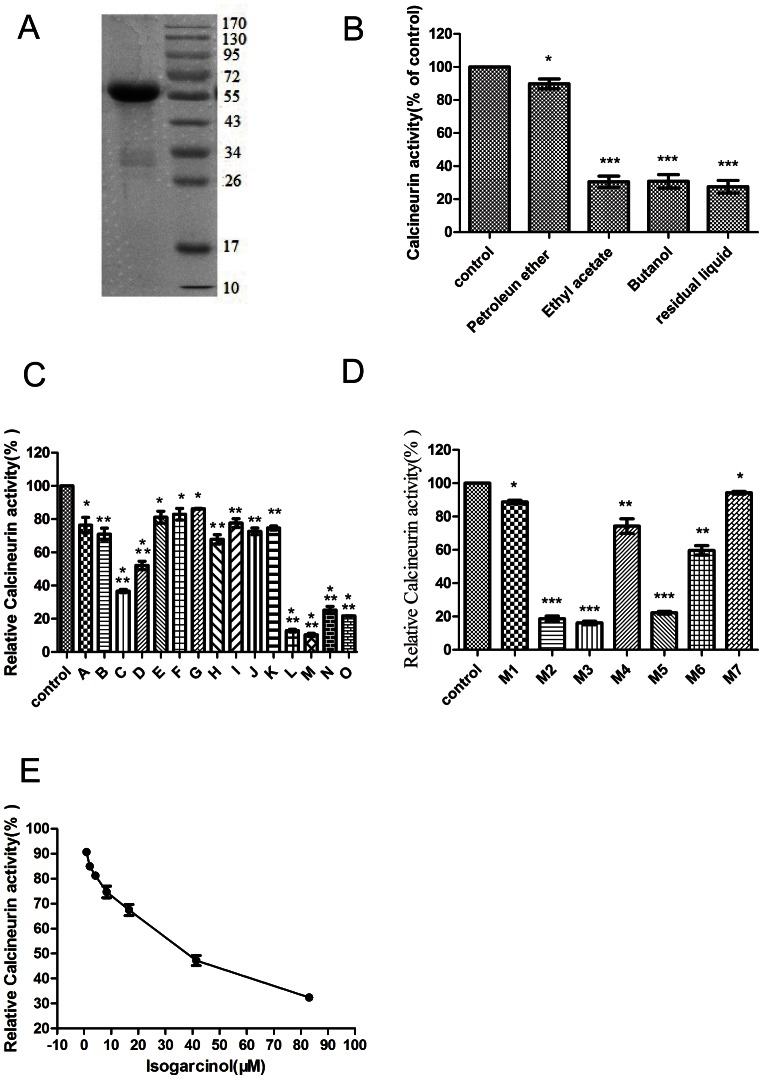
Inhibition of CN activity by *Garcinia mangostana* L. fractions. (A) SDS-PAGE analysis of purified CN protein. Lane 1: CNA; Lane M: Marker (B) Effects of *Garcinia mangostana* L. fractions on CN activity. (C) Effects of EtOAc fractions on CN activity. (D) Effects of fractions derived from fraction M on CN activity. (E) Inhibition of CN activity by compound I., Assays were performed with p-NPP as a substrate. CN activity is shown as a percentage of that in the control group. All values are expressed as mean ± SD. n = 3.

CNA was analyzed by SDS-PAGE and found to be electrophoretically pure (Figure 1A).

CN assays showed that the EtOAc and n-BuOH fractions and the residual liquid all inhibited CN activity (Figure 1B). Of the EtOAc fractions, fraction M was the most active (Figure 1C). Of the 7 fractions into which fraction M was further divided, the most active was fraction M3 (Figure 1D), which was recrystallized with 95% ethanol to yield compound I. After purification, 26.9 g of isogarcinol of 95.5% purity was obtained from 37.6 kg of *Garcinia mangostana* L. Hence the level of compound I present in *Garcinia mangostana* L. is 0.07%. Compound I inhibited CN at concentrations of 0–83.06 µM in a dose-dependent manner (IC50 = 36.35 µM, Figure 1E).

### Compound I identification

**Figure 2:**
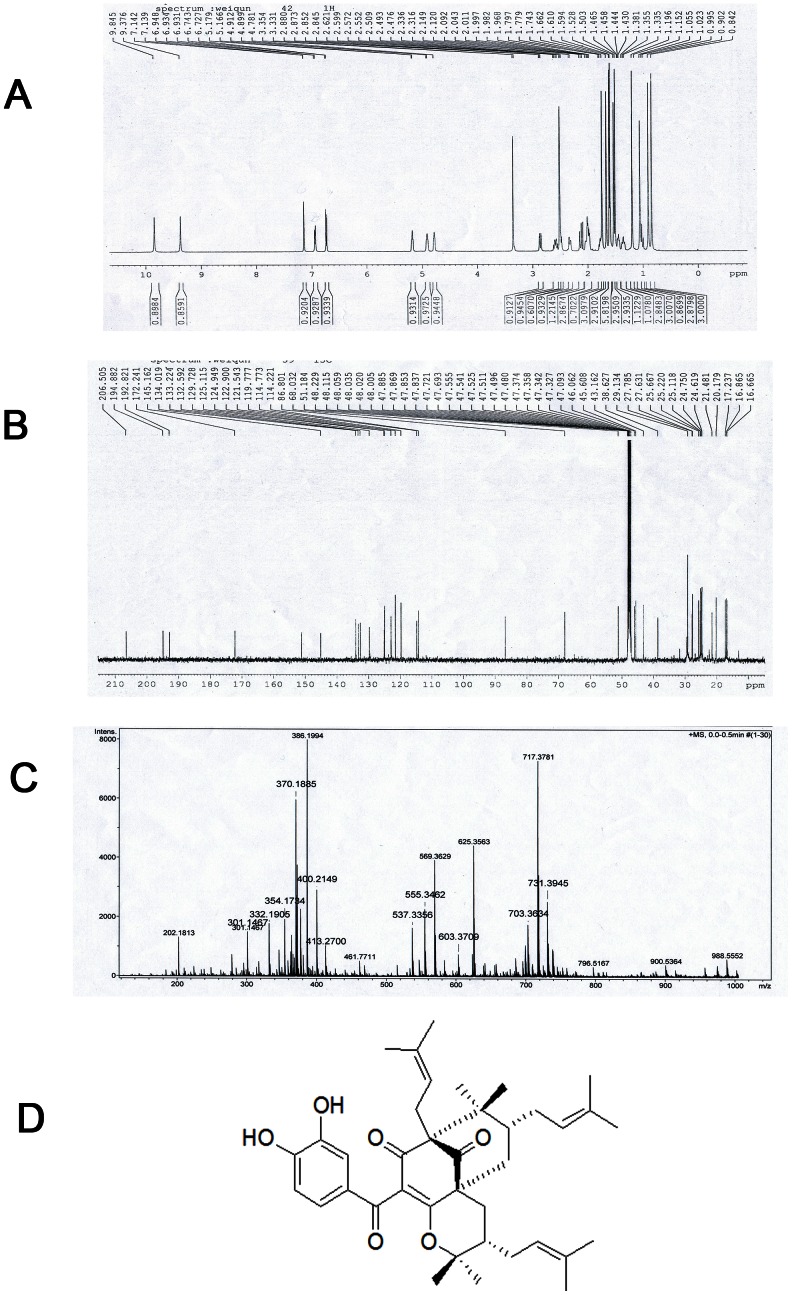
Compound I identification. (A) ^1^H-NMR spectrogram of compound I. (B) ^13^C-NMR spectrogram of compound I. (C) ESI-MS of compound I. (D) Chemical structure of isogarcinol.

Compound I was a colorless amorphous powder. NMR data for the compound were as follows. ^1^H-NMR [500 MHZ, DMSO] (Figure 2A) δ: 1.54 m (m, H-6, 1H), 2.28 (d, J = 14.5 Hz, H_a_-7, 1H), 2.06 (m, H_b_-7,1H), 7.30 (d, J = 2 Hz, H-12, 1H), 6.75 (d, J = 8.0 Hz, H-13, 1H), 7.16 (dd, J = 8.5, 2 Hz, H-14, 1H), 6.74 (d, J = 8.0 Hz, Hz, H-15, 1H), 7.16 (dd, J = 8.5, 2 Hz, H-16, 1H), 2.68 (m, H_a_-17), 2.45 (dd, J = 12.5, 5 Hz, H_b_-17, 1H), 4.87 (t, J = 5.6 Hz, H-18, 1H), 1.59 (s, H_3_-20, 3H), 1.61 (s, H_3_-21, 3H), 1.17 (s, H_3_-20, 3H), 1.00 (s, H_3_-20, 3H), 2.65 (m, H_a_-24, 1H), 2.20 (m, H_b_-24, 1H), 4.91 (t, J = 7.2 Hz, H-25, 1H), 1.66 (s, H_3_-20, 3H), 1.69 (s, H_3_-20, 3H), 3.05 (dd, J = 14, 3.5 Hz, H_a_-29, 1H), 1.02 (m, H_a_-29, 1H), 1.52 (m, H-30, 1H), 1.28 (s, H_3_-32, 1H), 0.92 (s, H_3_-33, 1H), 2.06 (m, H_a_-34, 1H), 1.80 (m, H_b_-34, 1H), 5.17 (t, H-35), 1.81 (s, H_3_-37, 3H), 1.59 (s, H_3_-38,3H).

^13^C-NMR [500 MHZ, MeOD] (Figure 2B) δ: 172.2 (C-1), 125.1 (C-2), 194.9 (C-3), 68.0 (C-4), 46.1 (C-5),45.6(C-6), 38.6 (C-7), 51.2 (C-8), 206.5 (C-9), 192.8 (C-10), 129.7 (C-11), 114.8 (C-12), 145.2 (C-13), 151.2 (C-14), 114.2 (C-15), 122.9 (C-16), 24.8 (C-17), 119.8 (C-18), 134.0 (C-19), 16.9 (C-20), 25.2 (C-21), 21.5 (C-22), 25.7 (C-23), 29.1 (C-24), 124.9 (C-25), 132.6 (C-26), 17.3 (C-27), 25.1 (C-28), 27.6 (C-29), 43.2 (C-30), 86.8 (C-31), 20.2 (C-32), 27.8 (C-33), 29.1 (C-34), 121.5 (C-35), 133.2 (C-36), 24.6 (C-37), 16.7 (C-38). ESI-MS m/z (%) (Figure 2C): 603.37 (M^+1^, 100), 625.36 (M^+23^, 100).

Comparison of the ^1^H and ^13^C-NMR spectral data with those reported in the literature [28] enabled determination of a partial structure. From this, compound I was identified as isogarcinol (Figure 2D).

### Inhibition of Con A-stimulated proliferation and MLR by isogarcinol

**Figure 3:**
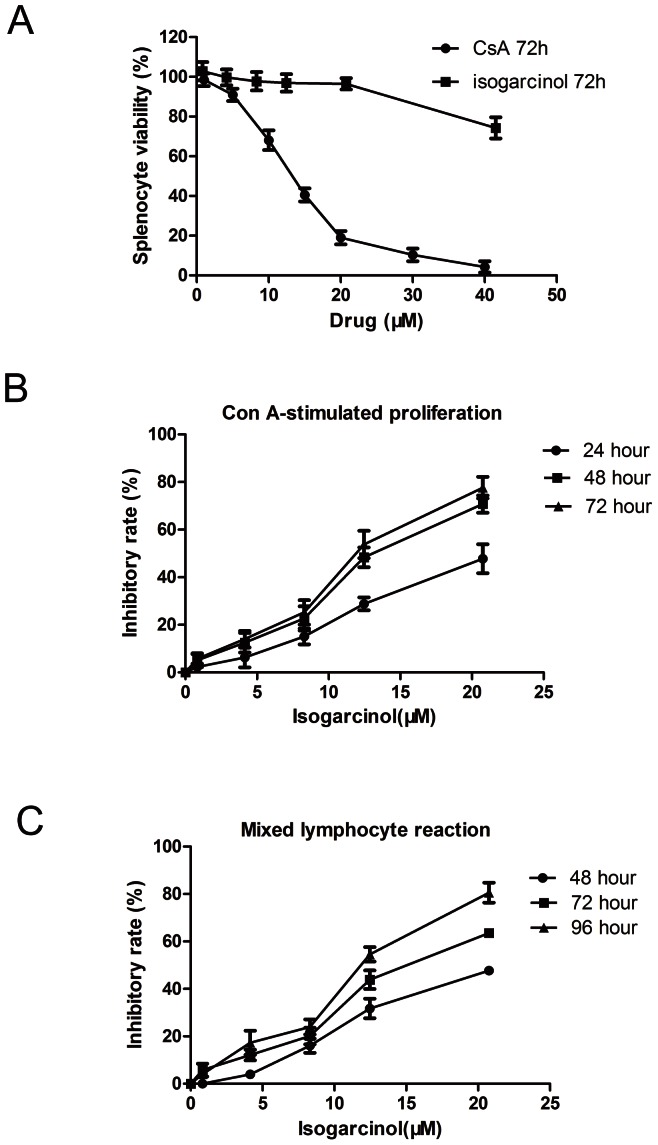
Inhibition of Con A-stimulated proliferation and MLR by isogarcinol. (A) Effect of isogarcinol on mouse splenocytes *in vitro*. Evaluation of different concentrations of isogarcinol and CsA on splenocyte viability, assessed by CCK-8 assay for 72 h. (B) Effect of isogarcinol at different concentrations on Con A-induced lymphocyte proliferation, assessed by CCK-8 assay for 24, 48 and 72 h. (C) Effect of isogarcinol at different concentrations on MLR, assessed by CCK-8 assay for 48, 72 and 96 h. Results are expressed as mean ± SD. n = 5 for each group.

The cytotoxicity of isogarcinol was tested in spleen lymphocyte cultures for 72 h. The results showed that isogarcinol at a concentration of 20.76 µM or lower had a small toxic effect on lymphocytes in the absence of mitogen in 72 h cell cultures, lower than that of CsA (Figure 3A). The immunosuppressive effects of isogarcinol were also analyzed using Con A-induced lymphocyte proliferation assays. In the lymphocyte proliferation assay, numbers of spleen lymphocytes in cultures stimulated with Con A for 24 h, 48 h and 72 h were 3.49±0.22, 5.57±1.09 and 8.93±0.92 times, those in control cultures, respectively. The IC50 values of isogarcinol for inhibition of Con A-induced T-lymphocyte proliferation for 24, 48, and 72 h were 30.25 µM, 15.00 µM, and 12.14 µM, respectively (Figure 3B). Similar results were found for the MLR: the IC50 values of isogarcinol for inhibition of the MLR for 48, 72, and 96 h were 24.89 µM, 18.99 µM and 11.27 µM, respectively (Figure 3C). Thus both Con A-induced lymphocyte proliferation and the MLR were suppressed by isogarcinol, and the inhibitory effect was dose- and time- related.

### Toxicity of isogarcinol in mice

Mice treated with isogarcinol (500 mg/kg and 2000 mg/kg) did not show any difference in gross general behavior, and there was no death during the fourteen days for which the mice were observed.

**Figure 4:**
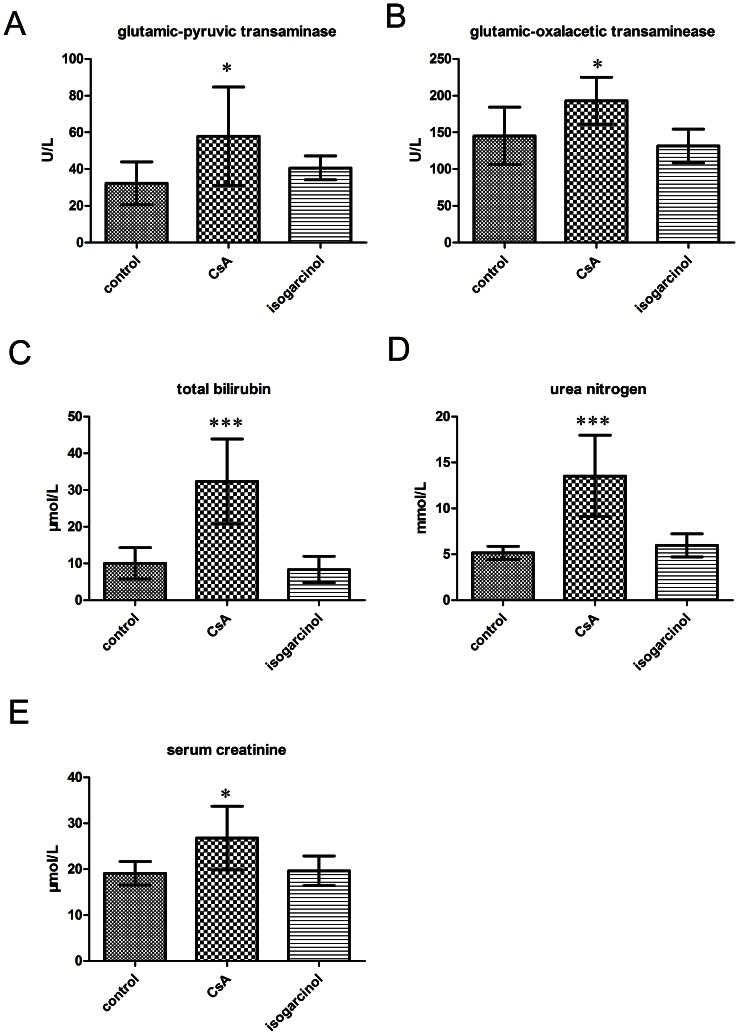
Toxicity of isogarcinol in mice. Blood levels of substances in BALB/c mice in control, CsA, and isogarcinol groups. n = 8 for each group. (A) Glutamic-pyruvic transaminase blood level. (B) Glutamic-oxalacetic transaminase blood level. (C) Total bilirubin blood level. (D) Urea nitrogen blood level. (E) Serum creatinine level.

Tests on the three groups of BALB/c mice (control, CsA and isogarcinol) showed that while blood biochemistry differed between the CsA and control groups, results for the isogarcinol group were similar to those for the control group, i.e. isogarcinol had less adverse effects than CsA in experimental animals (Figure 4).

### Inhibition of DNFB-induced DTH in mice by isogarcinol

**Figure 5:**
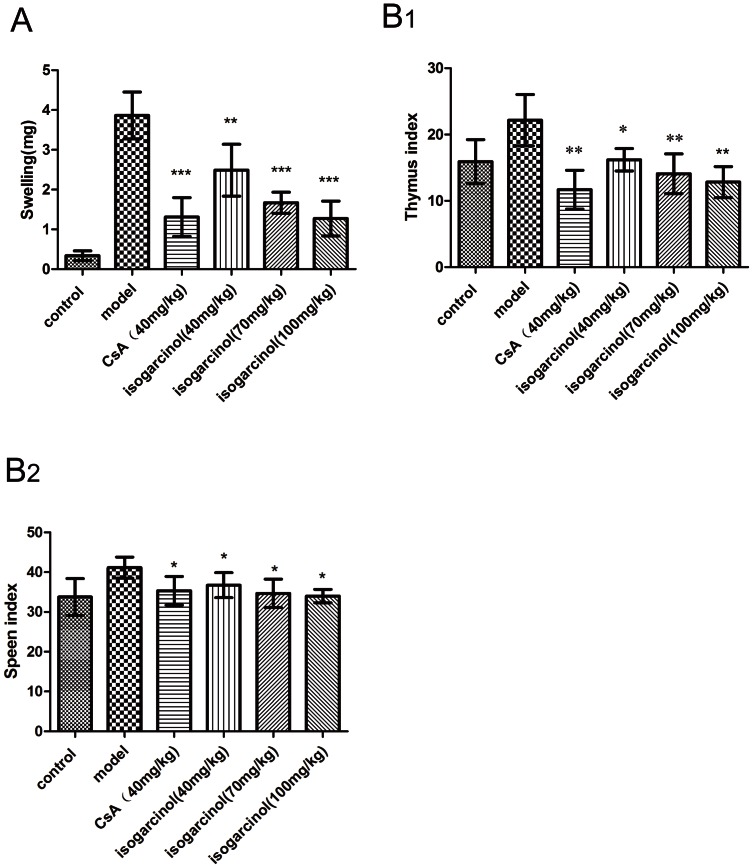
Inhibition of DNFB-induced DTH in mice by isogarcinol. Inhibitory effects of isogarcinol on DNFB-induced ear swelling in mice. (B_1_) Thymus index. (B_2_) Spleen index. Significant differences from the model group were evaluated using paired samples t-tests. *P<0.05, **P<0.01 and ***P<0.001.

Ear swelling was significantly higher in the model group than the control group (P<0.001), indicating that the DNFB-induced delayed-type hypersensitivity (DTH) model was successful. Ear swelling was significantly lower in mice treated with isogarcinol and CsA than in the model group (P<0.05) (Figure 5A) and isogarcinol had a dose-dependent effect on DNFB-induced DTH. Oral administration of 100 mg/kg isogarcinol had a similar effect on DTH to 40 mg/kg CsA.

The effects of isogarcinol on the spleen and thymus indexes of mice were also investigated. The results of the thymus index measurements paralleled the ear swelling (Figure 5B_1_), but there was no obvious effect of isogarcinol on the spleen index (Figure 5B_2_). This suggests that it has a strong inhibitory effect on the cellular immune response but not the humoral immune response. It was also observed that the fur of mice sensitized by DNFB became ruffled and the mice were emaciated, and oral administration of isogarcinol, but not CsA, relieved these symptoms.

### Improvement of allograft skin survival by isogarcinol

**Figure 6:**
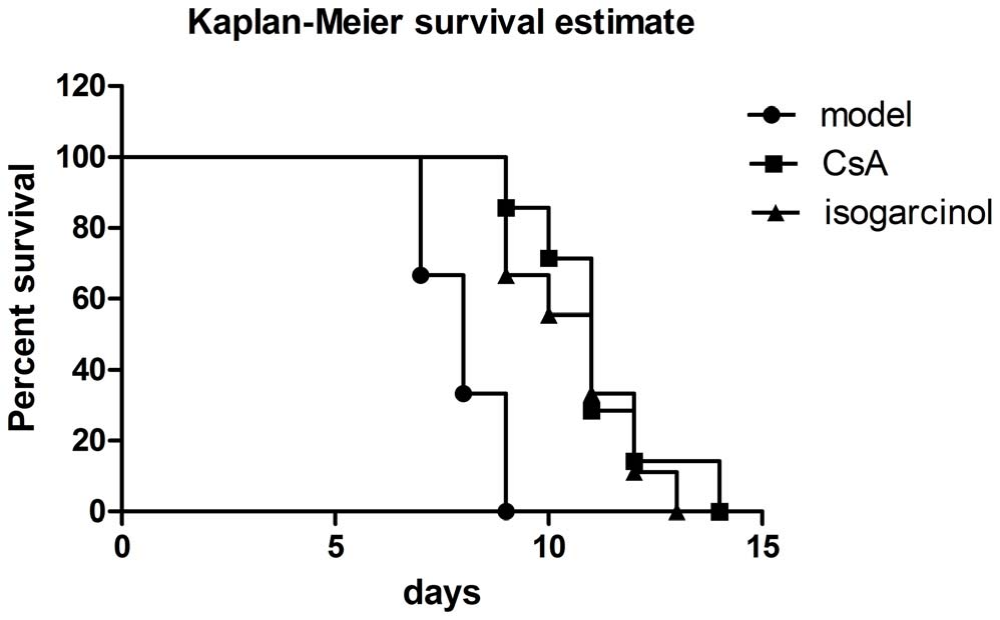
Prolongation of allograft skin survival by isogarcinol. Percentages of graft survival at different times after skin transplantation using Kaplan–Meier survival analysis.

Differences in skin graft survival were assessed using the Kaplan–Meier survival analysis. Survival time was significantly longer in mice treated with 100 mg/kg/day isogarcinol (10.66±1.50d) than in mice in the control model group (8.00±0.89d), and close to the survival time in mice treated with 40 mg/kg/day CsA (11.14±1.57d) (Figure 6). It was also observed that mice treated with CsA became restless, and emaciated, with ruffled fur, but that these symptoms did not occur in the mice treated with isogarcinol.

## Discussion

Traditional Chinese medicine (TCM) is a very rich source of potential new drugs. Although their clinical usage is limited by side effects such as nephrotoxicity and neurotoxicity, calcineurin inhibitors (CNIs) such as cyclosporin A and tacrolimus, are currently the most effective immunosuppressants, and are widely used to reduce rates of acute rejection and prolong graft survival in organ transplantation [13]. We aimed to discover, from TCM, novel, less toxic immunotherapeutic agents targeting calcineurin. In this study, we used CN as target enzyme to investigate the immunosuppressive properties of a series of natural compounds from *Garcinia mangostana* L. Ethyl acetate (EtOAc) and n-butanol (n-BuOH) extracts and the residual liquid inhibited calcineurin activity. The EtOAc fraction was first separated and purified using the CN enzymatic assay. After purification by two steps of silica gel chromatography, the bioactive polyisoprenylated benzophenone derivative, isogarcinol, was isolated from *Garcinia mangostana* L. Polyisoprenylated benzophenones have previously been isolated from plants, particularly of the Clusiaceae family, and their biological properties have received considerable attention from a pharmacological point of view in recent years [29]. Isogarcinol has been shown to possess anti-plasmodial [30], anti-bacterial [31], [32] and anti-cancer [33], [34] activities It also inhibits histone acetyltransferases [35]; dysfunction of these enzymes is known to lead to cancer.

In this study, we found for the first time that isogarcinol can inhibit calcineurin activity. Calcineurin plays an important role in immune regulation, suggesting that isogarcinol could have effects on immune reactions. Further analysis was consistent with this hypothesis.

Isogarcinol suppressed the proliferation of spleen lymphocytes induced by the T-cell mitogen Con A, and the expression of allotypic antigens in the MLR at concentrations that resulted in relatively low cytotoxicity. In the murine T-lymphocyte proliferation assay proliferation is induced by the T-cell mitogen Con A, so proliferation is fast and suppression is dramatic. However in the MLR, since the stimulators need to be treated with mitomycin C to generate the allotypic antigen, proliferation of the responders is slow and suppression is not very dramatic. Previous studies have shown that calcineurin plays a critical role in the process of T-lymphocyte activation [3]; it is reasonable to presume that isogarcinol displayed immunosuppressive activity *in vitro* mainly through its inhibitory effect on calcineurin.

The DNFB-induced DTH reaction is a Th1 cell-mediated pathologic response associated with T-cell activation and the production of many cytokines [36]. We found that isogarcinol strongly inhibited DNFB-induced ear swelling in a dose-dependent manner, demonstrating a suppressive effect on T-cell-dependent immune responses.

All allogeneic organ transplants are at risk of acute or chronic rejection. T-cells are central to the process of transplant rejection through allorecognition of foreign antigens, which leads to their activation and the orchestration of an effector response that results in organ damage [37]. The skin transplant model is a quick and easy method to monitor allogeneic T-cell responses and to evaluate the efficiency of immunosuppressive agents [38]. Isogarcinol significantly improved allogeneic skin graft rejection time in mice. It is reasonable to presume that isogarcinol displayed immunosuppressive activity *in vivo* mainly through its inhibitory effect on T-cells. Our results suggest that isogarcinol might protect against organ failure associated with transplantation of other organs.

Medicinal plants can serve as safer therapeutic alternatives. A large number of these plants and their isolated constituents have shown beneficial therapeutic effects including antioxidant, anti-inflammatory, anti-cancer, anti-microbial, and immunomodulatory effects [39]. Immunosuppressants have important clinical roles in organ transplantation and the treatment of autoimmune diseases. At present, the established immunosuppressants cyclosporin A (CsA), and tacrolimus (FK506), are widely used in organ transplantation. However, both drugs, which have similar mechanisms of action, have severe side effects, notably nephrotoxicity, neurotoxicity, hypertension and hyperlipidaemia [13], [40]. Thus, it is important to search for a novel class of immunosuppressive compounds with lower toxicity and fewer side effects. In this study, isogarcinol was tested for acute toxicity in mice within 14 days. No mice died during this period, even in the high dose group (2000 mg/kg), which indicates that the median lethal dose (LD_50_) of isogarcinol is greater than 2000 mg/kg. Thus the LD_50_ of isogarcinol is more than 50 times the effective dose of isogarcinol on DNFB-induced DTH in mice (40 mg/kg), which suggests that isogarcinol has low toxicity *in vivo* at the concentrations at which it has immune effects. It is well known that CsA displays hepatotoxicity; in most patients oral administration of large doses of CsA leads to increased total bilirubin and transaminase levels [41], [42]. In this study, we found that the levels of total bilirubin, glutamic-pyruvic transaminase, and glutamic-oxalacetic transaminase were increased in mice receiving large oral doses of CsA, but not in mice treated with isogarcinol. Considering the severe nephrotoxicity of CsA [13], we also investigated the levels of serum urea nitrogen and creatinine in mice treated with CsA and isogarcinol. Both were much higher in the CsA group than in the isogarcinol group. Overall, all the results showed that isogarcinol had fewer adverse effects in experimental animals It was also observed that, unlike CsA, isogarcinol could relieve the fur ruffling and emaciation in DNFB-induced DTH. Thus isogarcinol has much lower toxicity in animals than CsA.

Organ transplantation procedures in patients have increased due to the availability of effective immunosuppressant drugs, which prevent rejection of the transplanted organ and preserve graft function. Most patients need long-term medication to achieve this [43]. The low toxicity of orally administered isogarcinol in animal tests observed in this study suggests that isogarcinol could potentially serve as a novel oral immunomodulatory long-term medication for treatment of transplantation rejection and autoimmune diseases.

In conclusion, the results from this study establish that isogarcinol, an effective calcineurin inhibitor extracted from a natural source, exhibits low toxicity and compelling immunosuppressive effects, which are probably due to inhibition of T-cell functions. We believe that the data warrant further evaluation of isogarcinol as a potential oral immunomodulatory drug for treatment of transplantation rejection and autoimmune diseases. Both the prevention of organ transplant rejection and the treatment of autoimmune diseases are challenging clinical problems. Advances in immunosuppressants over the past decade have resulted in dramatic improvements in short- and long-term outcomes in organ transplantation and have reduced autoimmune disease [44]. With further chemical synthesis and modification, isogarcinol may well make a major contribution to future immunosuppression.
